# Assessing the role of mega cisterna magna in criminal responsibility: implications for neurocognitive disorders in forensic evaluations

**DOI:** 10.1186/s12888-025-06723-5

**Published:** 2025-03-25

**Authors:** Gamze Onar, Sena Inal Azizoglu, Ece Büyükakça, Fatih Oncu

**Affiliations:** 1 Bakirkoy Prof. Dr. Mazhar Osman Mental and Nervous Diseases Hospital Education and Research Hospital, Istanbul, Turkey; 2 Department of Psychiatry, Başakşehir Çam and Sakura City Hospital, Istanbul, Türkiye

**Keywords:** Mega cisterna magna, Criminal liability, Forensic psychiatry

## Abstract

This article explores the intersection of neurocognitive disorders and criminal behavior, highlighting the significant role of conditions such as traumatic brain injury (TBI), substance misuse, and neurodegenerative diseases in cognitive decline and associated criminal activities. We present three case studies of patients with mega cisterna magna diagnosed through imaging, discussing their legal proceedings and the impact of their cognitive impairments on criminal responsibility. The study underscores the prevalence of neurocognitive disorders among incarcerated and psychiatrically assessed individuals, suggesting these conditions are often underdiagnosed in forensic settings. Our findings indicate a critical need for comprehensive neurocognitive assessments to better understand and manage the influence of neurological disorders on behavior and legal responsibility. The cases demonstrate how neurocognitive impairments, particularly mega cisterna magna, can influence behavior and complicate the assessment of criminal responsibility, advocating for enhanced diagnostic practices and tailored treatment approaches in forensic psychiatry. This study calls for more focused research on neurocognitive disorders within forensic populations to refine diagnostic and treatment strategies, ultimately aiming to improve legal adjudications and clinical outcomes for affected individuals.

## Clinical trial number

Not applicable.

## Background

Neurocognitive disorders encompass a variety of conditions where the primary characteristic is a decline in cognitive abilities across multiple domains such as complex attention, executive functions, memory, language, perceptual motor skills, and social cognition [1]. These disorders are acquired rather than congenital and can stem from causes like traumatic brain injury, substance misuse, or neurodegenerative diseases. They frequently manifest alongside behavioral and emotional challenges, including impulsivity and emotional instability. In some instances, these issues may escalate to aggressive, violent, or criminal behaviors, including arson, theft, or sexual offenses [2, 3].

While often identified as an incidental neuroimaging finding, mega cisterna magna (MCM) typically does not require follow-up; however, recent research suggests potential associations between this condition and psychiatric disorders. MCM may occur in isolation or as part of the Dandy-Walker complex, a spectrum of posterior fossa malformations that includes varying degrees of vermian hypoplasia, cystic dilatation of the fourth ventricle, and enlargement of the posterior fossa [4, 5]. The Dandy-Walker complex is a significant anomaly linked to disruptions in embryological development, which may influence cerebrospinal fluid dynamics and neurodevelopment. Evidence increasingly highlights the potential role of cerebellar structural abnormalities, such as those seen in MCM or Dandy-Walker complex, in the pathophysiology of psychiatric conditions. This connection is demonstrated in a case report of a 36-year-old male with isolated MCM and shared psychotic disorder (folie à deux), highlighting the importance of investigating cerebellar neurodevelopmental abnormalities as potential predisposing factors in psychosis [6].

**Fig. 1:**
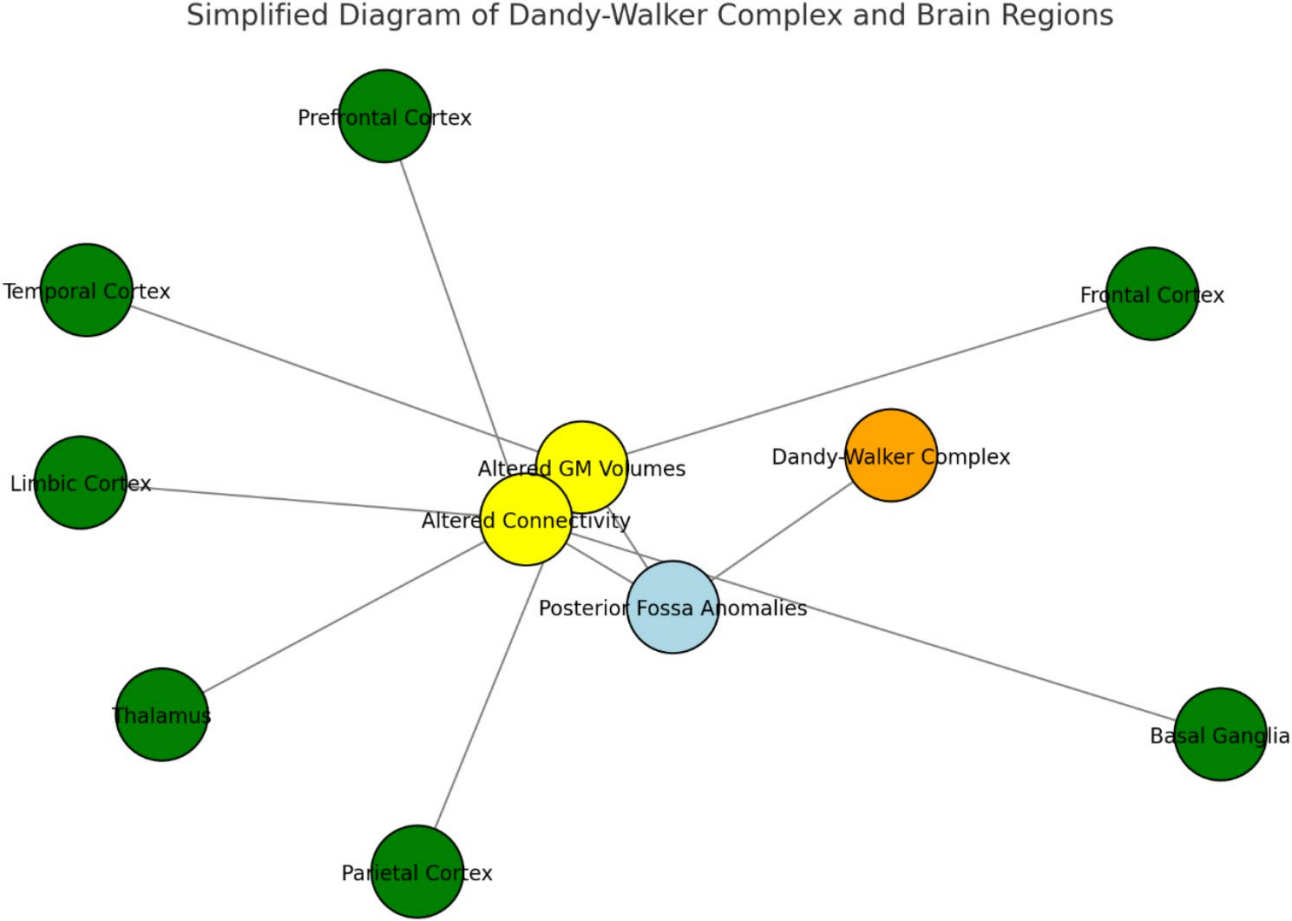
Diagram of dandy-walker complex and brain regions

The clinical manifestations of DWC vary widely depending on factors such as the severity of hydrocephalus, intracranial pressure, cerebellar-related motor and coordination impairments, and associated neurodevelopmental or behavioral comorbidities. Research indicates that reductions in cerebellar gray matter volume and disruptions in connectivity between the cerebellum and key brain regions—including the prefrontal, superior temporal, posterior parietal, and limbic cortices—may underlie deficits in social functioning, language, repetitive behaviors, cognitive processes, and emotional regulation. These disruptions are implicated in various neuropsychiatric disorders, including autism spectrum disorder (ASD), attention-deficit/hyperactivity disorder (ADHD), schizophrenia, and mood disorders. This highlights the critical role of cerebellar development in both motor and neuropsychological outcomes associated with DWC. In forensic psychiatry, evaluating structural brain anomalies are essential. Neuroimaging techniques such as MRI help identify conditions like MCM, while electroencephalography (EEG) can rule out seizure activity that may present similarly to psychiatric conditions. Furthermore, neuropsychological assessments (e.g., IQ tests, executive function tasks, personality inventories) provide a more nuanced understanding of an individual’s cognitive and affective status, helping inform both clinical management and legal decisions [7–9] (Fig. 1).

Evidence linking neurocognitive disorders with criminal behavior is substantial and comes from both cross-sectional and longitudinal studies. Research, including a systematic review of 16 studies, supports the connection between early-life TBI and later antisocial or criminal actions [10]. Similarly, conditions such as dementia are linked to a higher likelihood of various offenses [11]. Additional indirect evidence comes from prevalence studies in prisons and forensic psychiatric settings, indicating that conditions like dementia are significantly more common among incarcerated older adults compared to the general population, with some studies reporting that up to 40% of elderly forensic psychiatric patients suffer from cognitive impairments including dementia or TBI [12, 13].

In Turkish Criminal Law, criminal responsibility is assessed based on the perpetrator’s ability to comprehend the nature of their act and regulate their behavior (Article 32 of the Turkish Penal Code). When cognitive or mood disturbances associated with mega cisterna magna impair these abilities, questions arise regarding potential reductions in criminal responsibility. Although illegal behaviors have been observed in neurodegenerative disorders, data on its prevalence remain limited and further studies are needed to examine this aspect of the disease more comprehensively. In this article, we discuss the criminal liability of three patients who were found to have mega cisterna magna on imaging.

## Case series

### Case 1

**Fig. 2:**
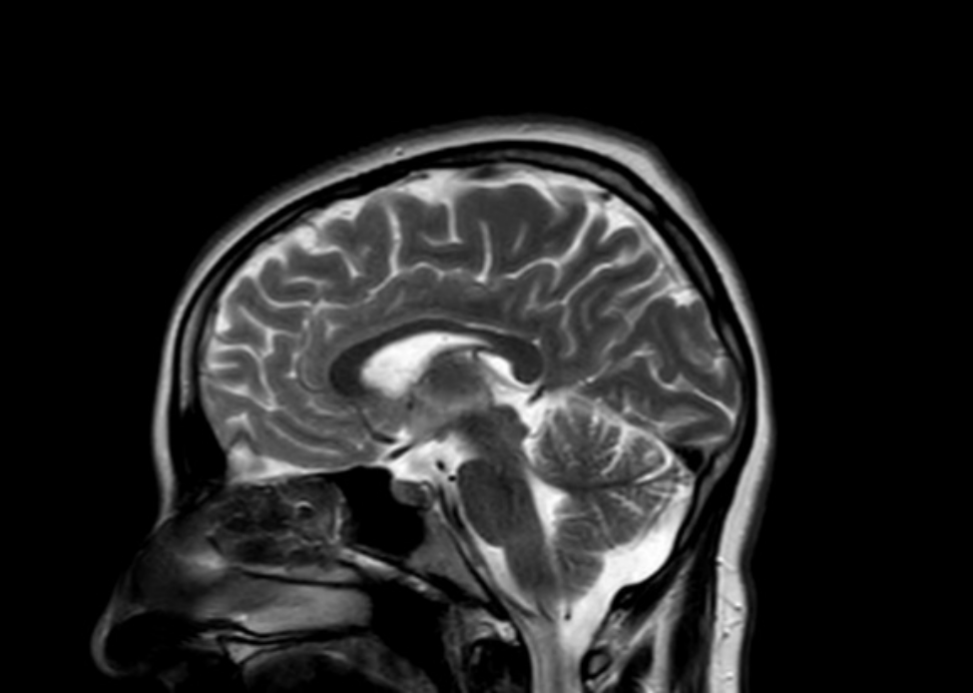
MRI image of case 1

The patient, a 21-year-old born in Beyoğlu, is a high school graduate and an only child living with his mother following his father’s death due to myocardial infarction. His father had a history of bipolar disorder. In 2019, he attempted suicide using medication. He does not use tobacco, alcohol, or drugs. During the mental status examination, he was conscious, oriented, and cooperative but reluctant to engage. His affect was restricted, mood was euthymic, and both his speech rate and volume were decreased. His associations were scattered, and due to his dissociative attitude, it was difficult to assess his thought content. His reasoning was impaired, and he lacked insight; no active homicidal or suicidal thoughts or plans were present. An Electroencephalogram (EEG) could not be conducted due to lack of cooperation. MRI results revealed a mega cisterna magna (Fig. 2). His Proteus score was 71 and his Verbal IQ was measured at 92. He was uncooperative during Neuropsychological Testing (NPT). The Rorschach test indicated psychotic features. His scores were 0 on the Young Mania Rating Scale, 27 on the Hamilton Depression Rating Scale, and 104 on the Positive and Negative Syndrome Scale (PANNS). He was tried for the offense of insult on July 3, 2020, and his criminal responsibility was removed because the patient’s diagnosis of Atypical Psychosis (alongside mega cisterna magna) and associated cognitive-psychotic features significantly impaired his ability to understand and control his actions.

## Case 2

**Fig. 3:**
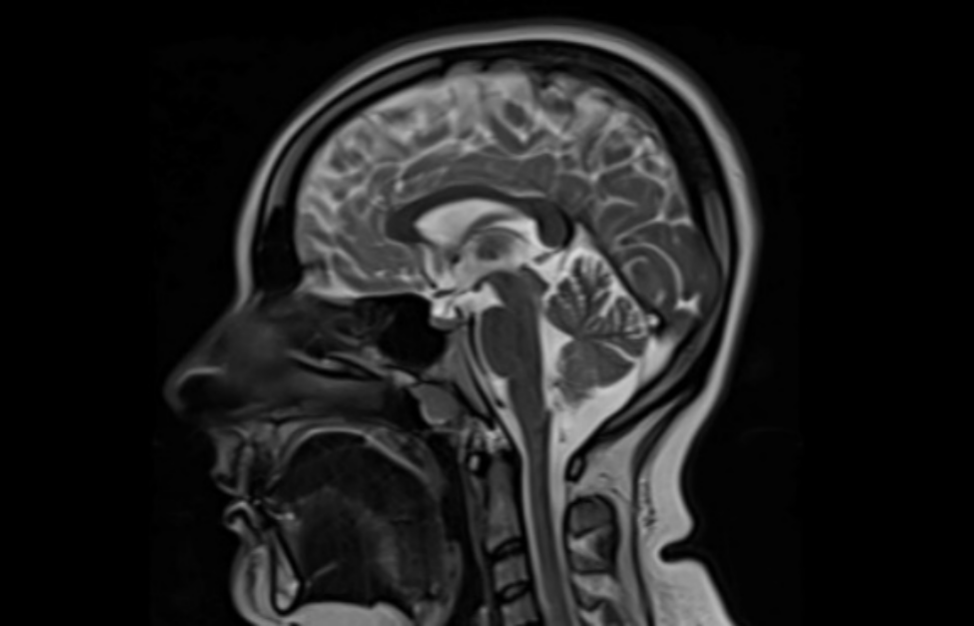
MRI image of case 2

The 27-year-old patient lives with his parents and four siblings in Yalova. He is a high school graduate, not preparing for university entrance exams, and is single with no children or marriage history. He has been using Olanzapine 20 mg and Quetiapine 200 mg daily since 2021 for a diagnosis of Bipolar Disorder and has been hospitalized at least once. EEG results are normal, and an MRI has revealed a mega cisterna magna (Fig. 3). His Wechsler Adult Intelligence Scale (WAIS) score is 93. The Rorschach test, while limited in data, highlights difficulties in reasoning and interpersonal relationships characterized by insecurity, immaturity, and suspicion. Neuropsychological Testing results show that other cognitive functions are normal, with mild verbal and non-verbal memory impairments and moderate findings related to the frontal axis. This memory impairment is characterized by impaired recall in the frontal type, though the recognition phase is normal. Minnesota Multiphasic Personality Inventory (MMPI) results do not show any significant psychopathology due to a defensive attitude, but histrionic features are prominent. He does not use tobacco, alcohol, or drugs. During the initial examination, he was conscious, oriented, and cooperative; eager to engage and respectful. His affect was restricted, mood was euthymic, and both speech rate and volume were normal. His associations were regular with no active psychotic signs. His reasoning was healthy, insight adequate, and there were no active homicidal or suicidal thoughts or plans. His scores were 0 on the Young Mania Rating Scale, 19 on the Hamilton Depression Rating Scale, and 57 on the Positive and Negative Syndrome Scale. In 2021, his criminal responsibilty was revoked due to the patient’s schizoaffective disorder (alongside mega cisterna magna), which compromised his judgment and prevented him from fully appreciating the nature of his behavior.

## Case 3

**Fig. 4:**
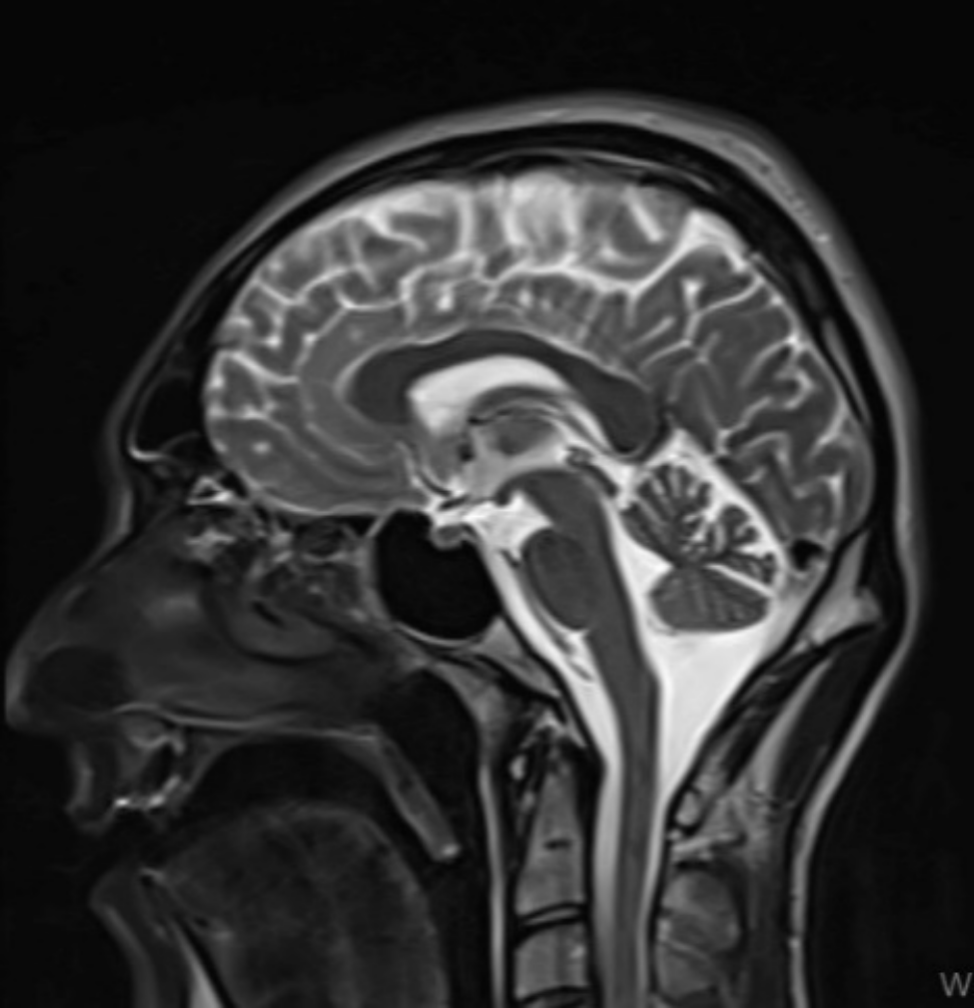
MRI image of case 3

The patient, a 28-year-old, lives with his parents and siblings. He was educated up to the 8th grade of primary school and repeated grades three times. He was exempted from military service due to intellectual disability. In September 2024, the patient faced legal proceedings for charges of threats and insults, which coincided with his first psychiatric admission. There are indications that his parents might also have limited mental capacities. During the examination, the patient was conscious, oriented, and cooperative but reluctant to participate in the interview. He exhibited restricted affect and a mildly irritable mood with reduced speech rate and volume. His thought content was poor, associations were regular, and reasoning abilities were impaired. He lacked insight and had no active homicidal or suicidal ideation or plans. EEG results were normal, and brain MRI revealed a mega cisterna magna (Fig. 4). His WAIS score was measured at 67, and the Rorschach test indicated limited mental capacity. The patient has a history of using psychoactive substances, including synthetic cannabinoids. Scores were 8 on the Young Mania Rating Scale, 29 on the Hamilton Depression Rating Scale, and 73 on the PANSS. Based on all these findings, criminal responsibility was diminished because the patient’s intellectual disability and limited mental capacity (alongside mega cisterna magna) hindered his capacity to comprehend the consequences of his actions.

## Discussion

Research concerning the prevalence of neurocognitive disorders in individuals assessed for criminal responsibility within psychiatric settings is remarkably sparse. Studies such as the one conducted by Fazel et al. reveal that the prevalence figures for psychiatric disorders observed in prison populations are substantially lower when derived from routine clinical evaluations as opposed to those obtained via structured diagnostic tools [14]. This discrepancy leads to a hypothesis that neurocognitive disorders are likely underdiagnosed among individuals within the criminal justice system.

In light of the possible influence of cerebellar and related cortical pathways, we posit that MCM could impair criminal responsibility through mechanisms such as decreased inhibitory control, heightened impulsivity, and compromised executive functioning [15]. These dysfunctions may manifest as impaired judgment, difficulty distinguishing right from wrong, or exacerbation of psychiatric symptoms—particularly in individuals already vulnerable to serious mental illness. Consequently, MCM may act as a contributory neuroanatomical factor that interacts with other psychiatric or cognitive impairments, intensifying their impact on criminal responsibility. Recognizing these potential mechanisms underscores the need for thorough neurocognitive assessments and multimodal evaluations, helping to ensure that legal and clinical decisions accurately account for the interplay between MCM and co-occurring psychiatric conditions.

Recent genetic studies suggest that certain genetic factors may increase the risk for schizophrenia, bipolar disorder, and schizoaffective disorders, indicating potential shared underlying mechanisms [16]. While the exact causes of conditions like mega cisterna magna, mania, or schizophrenia remain unclear, research by Langarica et al. in 2005 proposed that both psychotic disorders and mega cisterna magna could stem from a common neurodevelopmental abnormality [17].

The connection between cerebellar anomalies and psychosis was first highlighted in 2001, when Turner et al. described a case of schizophrenia-like psychosis in a person with a Dandy-Walker variant and vermian hypoplasia [18]. Langarica and Peralta later suggested that even milder cerebellar abnormalities, like mega cisterna magna—which typically spares the vermis—may still be linked to psychosis. Supporting this, MRI studies have shown that some degree of vermian dysgenesis is present across all forms of the Dandy-Walker complex, including mega cisterna magna. These findings highlight the importance of cerebellar development in understanding the potential connections between structural brain anomalies and psychiatric conditions [19].

In forensic psychiatric practice, where the primary focus is often the stabilization of acute psychiatric symptoms—including psychosis, severe mood disorders, and acute behavioral crises like severe aggression and self-harm—there is a risk that underlying neurocognitive disorders may be overlooked [20]. This oversight can occur despite the potential of such disorders, including anatomical variations like mega cisterna magna, to significantly impact cognitive and behavioral functions. Mega cisterna magna, which involves an enlargement of the cerebrospinal fluid spaces near the cerebellum, could influence a range of neurological functions potentially linked to criminal behavior [21]. Accurate recognition and understanding of the role of such neurocognitive impairments could be crucial in the initial stabilization phase of treatment. A robust body of research indicates that interventions for behavioral disturbances in conditions like dementia should be highly individualized and thoroughly assessed, prioritizing specific non-pharmacological interventions [22].

Moreover, the pharmacological management of symptoms such as agitation in patients with traumatic brain injuries can involve a tailored use of medications like propranolol, methylphenidate, valproic acid, and the antipsychotic olanzapine. These treatment modalities underscore the necessity for a nuanced approach to the pharmacotherapy of neurocognitive disorders [22].

The Risk-Need-Response (RNR) model is a widely recognized framework in forensic psychology that aims to reduce recidivism by aligning interventions with an individual’s specific risk factors, criminogenic needs, and responsiveness. In the context of MCM, this model emphasizes the importance of addressing neurocognitive deficits that may impair an individual’s ability to benefit from standard treatment or rehabilitation programs. As discussed in our case series, MCM can be associated with mood and cognitive impairments, potentially compounding risk factors for criminal behavior. By recognizing these deficits early and tailoring interventions, such as cognitive-behavioral strategies or specialized neurorehabilitation, to an individual’s unique clinical profile, practitioners can reduce the impact of MCM on criminal responsibility. This approach allows for effective management of underlying neurocognitive deficits and ultimately contributes to more accurate risk assessments, more targeted treatments, and improved outcomes in forensic settings [23].

Despite increased awareness and efforts toward cross-cultural neuropsychological assessment in Europe over recent decades, there remains a considerable gap in both the knowledge among clinicians and the availability of suitable neuropsychological tests for diverse populations [24]. Future research initiatives should focus on prospectively screening for neurocognitive disorders among forensic psychiatric patients, followed by comprehensive neurological and neuropsychological assessments to accurately establish the prevalence of these disorders in this unique population. These studies should also aim at developing and refining best practices for treating such patients within the forensic context, possibly by adapting successful treatment strategies from general psychiatric settings to the specific needs of forensic populations [25].

Existing literature on treatment within forensic psychiatry shows a significant void specifically concerning the management of neurocognitive disorders, suggesting an urgent need for research that tests the efficacy and applicability of established psychiatric practices in the forensic setting [26]. Challenges such as a lack of neuropsychological expertise or logistical difficulties in conducting necessary neuroimaging studies, such as MRI scans at external facilities, need to be identified and addressed to improve the quality of care for these patients [27].

Moreover, the data indicate that brain pathology scores are significantly higher among forensic inpatients compared to healthy non-criminal controls, and about half of the forensic patients display signs of brain pathology. This finding supports the necessity of including comprehensive assessments of brain damage in forensic psychiatric evaluations. It is often the case, especially in jurisdictions like Germany, that adequate neuroradiological evaluations are not performed routinely, despite the potential for such assessments to support legal defenses such as insanity pleas or to guide treatment planning. As clinical experience suggests, patients with organic brain damage often present with treatment-resistant mental health issues and do not respond as effectively to conventional therapies, such as cognitive-behavioral therapy, as do patients with primarily nonorganic mental health disorders [28].

### Limitation

A notable limitation of this study is the absence of direct examination of specific neuroanatomical variations like mega cisterna magna, which may impact cognitive and behavioral functions relevant to criminal responsibility. This gap highlights the need for more focused research incorporating detailed neuroimaging to determine the influence of such anatomical features on forensic evaluations.

## Conclusion

This study underscores the critical need for systematic neurocognitive assessments in forensic settings to better understand and address the influence of neurological disorders like mega cisterna magna on criminal responsibility, ultimately enhancing legal adjudications and treatment interventions for affected individuals.

## Data Availability

No datasets were generated or analysed during the current study.
